# Low Drug Loading Hampers the Clinical Translation of Peptide Drugs-Containing Metered-Dose Inhalers

**DOI:** 10.3390/ph15040389

**Published:** 2022-03-23

**Authors:** Zhengwei Huang, Lei Shu, Ying Huang, Chuanbin Wu, Xin Pan

**Affiliations:** 1 College of Pharmacy, Jinan University, Guangzhou 510632, China; huangzhengw@jnu.edu.cn (Z.H.); shulei@stu2021.jnu.edu.cn (L.S.); chuanbin_wu@126.com (C.W.); 2 School of Pharmaceutical Sciences, Sun Yat-sen University, Guangzhou 510006, China

**Keywords:** peptide drugs, pulmonary administration, MDI, drug loading, amphiphilic materials

## Abstract

Peptide-based drugs have attracted extensive attention from the medical and pharmaceutical industry because of their relatively high safety and efficacy. However, most of the peptide drugs approved are administrated by injection, which can easily cause poor patient compliance. In this circumstance, pulmonary administration as an alternative to injection administration can not only avoid the above issue but also accelerate the absorption rate of peptide drugs and improve bioavailability. Among the pulmonary delivery systems available on the market, metered-dose inhalers (MDIs) have emerged as appealing candidates for pulmonary delivery systems with clinical translational value, owing to their many merits, including portable, easy-to-operate, and cost-effective properties. Nevertheless, the industrialization of peptide drugs-containing MDIs encounters a bottleneck of low drug loading, owing to the incompatibility between the propellant and the peptide drugs, which cannot be effectively overcome by the current carrier particle encapsulation strategy. Herein, we put forward the following strategies: (1) To screen amphiphilic materials with high surface activity and strong interaction with peptide drugs; (2) To construct a chemical connection between peptide drugs and amphiphilic substances; (3) To optimize the cosolvent for dispersing peptide drugs. We suppose these strategies have the potential to defeat the bottleneck problem and provide a new idea for the industrialization of peptide drugs-containing MDIs.

## 1. Pressing Need for Developing New Delivery Approach for Peptide Drugs

Peptide drugs are defined as the peptide species with specific pharmacodynamic activity, which can be used in the clinical therapy of various diseases [1]. Compared with chemical drugs, most peptide drugs are natural or endogenous products that show satisfactory biosafety [2]. Over the past several decades, the safety concern of chemical drugs has been growing. In contrast, peptide drugs have been attracting the clinical and pharmaceutical industry due to their relatively high safety and effectiveness. According to the literature, there are approximately 80 peptide drugs on the market, 170 in clinical trials and >200 under preclinical development [3,4]. The marketing scales exceeded USD 14.7 billion, and the annual growth rate reached 20% [5]. It can be concluded that peptide drugs have a huge market potential, which motivates the continuous research interests in this field.

Peptide drugs are generally unsuitable for oral administration because they are commonly vulnerable to the enzymes in the gastrointestinal tract. It is reported that less than 10% of the approved peptide drugs are intended for oral, nasal, and transdermal delivery [6], while over 90% of them are delivered through injections [7]. Injection usually is the least acceptable administration route for patients owing to injection pain. Worse still, the peptide drugs approved are mainly intended for the treatment of long-term diseases (diabetes, tumors, etc.) [8], and the long-term injection will induce trauma and infection, which cause poor patient compliance [9]. In addition, the medical resources will be substantially consumed during the long-term injection. Consequently, the current administration route limits the further clinical application of peptide drugs; it is urgent to exploit a new administration route for peptide drugs to meet the clinical demands.

In order to achieve desired drug administration route, multiple novel drug delivery systems for peptide drugs delivery have been designed and developed: New generation injections, tempered oral delivery systems, microneedling delivery systems, pulmonary delivery systems, etc. New generation injections consist of nanoassemblies with targeting or controlled release behaviors [10,11,12,13], and thus the frequency of injections can be substantially reduced. However, as an invasive delivery route, they still pose needle phobia in patients. As mentioned above, oral delivery is a widely accepted administration route; tempered oral delivery systems can minimize the degradation of peptide drugs in the gastrointestinal tract by the protecting materials [14], which are highly promising delivery systems for peptide drugs. Nevertheless, the physiological barriers in the gastrointestinal tract render a low and variable absorption of peptide drugs [15], leading to unsatisfactory bioavailability of oral-delivered peptide drugs. Microneedling delivery systems have been burgeoning recently, which are (almost) noninvasive systems that exhibit encouraging pharmacokinetics profile in preclinical studies [16]. It should be noted that these systems are mainly under laboratory investigation, and there is a long way towards clinical translation.

## 2. Metered-Dose Inhalers Are Promising Alternatives for Peptide Drugs Delivery

Amongst the aforementioned systems, the pulmonary delivery system is a promising alternative for peptide drugs administration. There is a tremendous capillary network and epithelial area in the lung region [17], accelerating the absorption rate of peptide drugs; the low enzymatic activity and bypassing the first-pass effect prevent the degradation of peptide drugs. Consequently, the drug bioavailability is high, which is comparable to that of injections [8]. As a noninvasive administration route, pulmonary delivery can avoid needle phobia, trauma, and infection, significantly improving patient compliance. Additionally, the concept of pulmonary delivery is relatively mature, and the commercialization is expectable.

Four kinds of pulmonary delivery systems are available on the market, viz. nebulizers, dry powder inhalers (DPIs), soft mist inhalers, and metered-dose inhalers (MDIs) [18]. Nebulizers should be used under the care of professional staff and are not portable drug delivery devices [19]. The research and development (R&D) processes of DPIs and soft mist inhalers are quite costly. MDIs possess portable, easy-to-operate, and cost-effective advantages compared with the other systems [20], which have been employed in medicine since the 1950s [21] and are considered as a mainstream pulmonary delivery system.

Taken together, MDIs have emerged as an appealing pulmonary delivery system candidate with clinical translational value.

## 3. Bottleneck Issue of Clinical Translation for Peptide Drugs-Containing Metered-Dose Inhalers

### 3.1. Low Drug Loading: The Bottleneck Issue

Despite the abovementioned advantages, up until now, no peptide drugs-containing MDIs have been translated from bench to bedside. It is known that peptide drugs-containing DPIs have reached the marketing process, e.g., the insulin-containing DPI (Afrezza^®^, MannKind, Russell Ranch Road, Westlake Village, CA, USA). However, the cost for these products is typically high. If an MDI system was developed to load insulin, maybe the product cost could be further decreased. What hinders the industrialization of peptide drugs-containing MDIs?

The authors argue that the industrialization of peptide drugs-containing MDIs is encountering a bottleneck issue—low drug loading. Such an issue undermines the strengths of peptide drugs-containing MDIs, raising the risk in R&D. The underlying reason for low drug loading should be determined in order to settle the bottleneck issue and boost the clinical translation of related products.

### 3.2. The Cause for the Bottleneck Issue

A fundamental reason for low drug loading is the incompatibility between the propellant of MDIs and peptide drugs. The propellant is a critical and indispensable excipient in MDIs, acting as the atomizing driving force and the dispersion matrix. The approved and widely used propellants include 1, 1, 1, 2-tetrafluoroethane (encoded as HFA 134a) and 1, 1, 1, 2, 3, 3, 3-heptafluoropropane (encoded as HFA 227). For clarity, the basic information for HFA 134a and HFA 227 is summarized in Table 1.

Although propellants exhibit a certain degree of water solubility, they are hydrophobic materials in nature [24]. In addition, most peptide drugs are hydrophilic molecules. Those mentioned above are the possible reasons for low compatibility between propellants and peptide drugs. It is difficult to homogeneously mix propellants and peptide drugs. Also, peptide drugs should interact with functional groups (mainly hydrogen bonding sites) of the solvent molecules to maintain conformational stability [25]. Propellants cannot interact with peptide drugs without functional groups, which leads to the unstable conformation of peptide drugs in propellants [26]. Consequently, if directly loaded in propellants, peptide drugs cannot adjust the conformation and tend to aggregate, eventually resulting in the destruction of the system stability.

### 3.3. Established Technology Cannot Well Overcome the Bottleneck Issue

To surmount the incompatibility, direct contact between propellants and peptide drugs must be avoided. To this end, pharmaceutical scientists put forward a strategy, which can be named the carrier particle encapsulation strategy. In most cases, the carrier refers to amphiphilic materials with a hydrophilic domain and a hydrophobic domain. The hydrophilic domain with functional groups can interact with the peptide drugs, while the hydrophobic domain shows a high affinity to the propellants. The amphiphilic materials can self-assemble into carrier particles in peptide drugs-containing MDIs, and this is generally accomplished at a high concentration. Importantly, the carrier particles serve as a ‘buffer zone’ preventing direct contact between propellants and peptide drugs.

The representative studies using the carrier particle encapsulation strategy are listed in Table 2. Poly(lactic-co-glycolic acid) (PLGA) was prepared into porous microspheres to incorporate bovine serum albumin [27]; nanoemulsion system was constructed by poloxamer as emulsifier and ethanol as coemulsifier to encapsulate exenatide [28] and salmon calcitonin [29]; insulin was loaded in lecithin–lactose carrier particles [30,31]; the feasibility of incorporating salmon calcitonin in anhydrous lecithin carrier particles was investigated [32]. These systems revealed encouraging outcomes in in vitro or in vivo studies, and detailed information can be found in Table 2.

It is well recognized that the widely applied carrier particle strategy can mitigate the incompatibility between propellant of MDIs and peptide drugs because the carrier materials can act as the ‘buffer zone’. Of note, the drug loading of the reported MDIs was still not sufficient for clinical use. The concentration of peptide drugs was within the range of 0.047~0.396 mg/mL, and the delivered dose per spray was 1.6~39.6 μg (Table 2). Nevertheless, Table 2 shows that the dose (per day or per use) of most peptide drugs exceeds 40 μg. If setting one spray for the regular therapeutic regimen, the drug loading of previously constructed peptide drugs-containing MDIs cannot satisfy the clinical use.

Two plausible solutions are using a spacer or more than one spray. The use of a spacer needs extra patient training [35], which may reduce patient compliance; the use of more than one spray will induce actuation–inhalation incoordination [36], significantly increasing the administration difficulty. Therefore, these are not appropriate remedies. Regretfully, the bottleneck issue still exists, and continuous efforts are needed to overcome it.

The authors infer that the low proportion of peptide drugs in the carrier particles is the direct cause of the low drug loading. Seen from Table 2, it is found that the proportion of amphiphilic materials in the carrier particles was up to 91~98%, while that of peptide drugs was lower than 9% (mostly lower than 5%). The reason for this fact is that a large number of amphiphilic materials should be used to construct the ‘buffer zone’, or an effective barrier between propellants and peptide drugs cannot be established.

Accordingly, most inner space of the carrier particles was occupied with amphiphilic materials, and the peptide drugs were only provided with minor accommodation. Thus, the drug loading in a single carrier particle was low. The total drug loading can be expressed as

(1) $$W_{\mathrm{total}}=n\cdot W_{\mathrm{single}}$$

where *W*_total_, *n*, and *W*_single_ represent the total drug loading, macroscopical particle concentration, and the drug loading in a single particle, respectively. Because the macroscopical particle concentration should be controlled below the threshold of particle aggregation [37] (viz. *n* is restricted), the low proportion of peptide drugs (meaning low *W*_single_) would finally lead to a low total drug loading (*W*_total_). It should be noted that the number of amphiphilic materials cannot be significantly lowered (*W*_single_ is restricted); that is, *W*_total_ of carrier particle strategy-based peptide drugs-containing MDIs is limited.

In summary, the low drug loading of peptide drugs-containing MDIs is originated from the incompatibility between propellants and peptide drugs, and the currently adopted carrier particle strategy can only mitigate the incompatibility issue but cannot effectively enhance the drug-loading capacity.

## 4. Possible Strategies for Enhancing the Drug Loading

As stated above, the insufficient proportion of peptide drugs in carrier particles resulted in the poor drug loading of peptide drugs-containing MDIs. In view of this, elevating the proportion of peptide drugs in carrier particles plays a pivotal role in improving drug loading.

**Figure 1:**
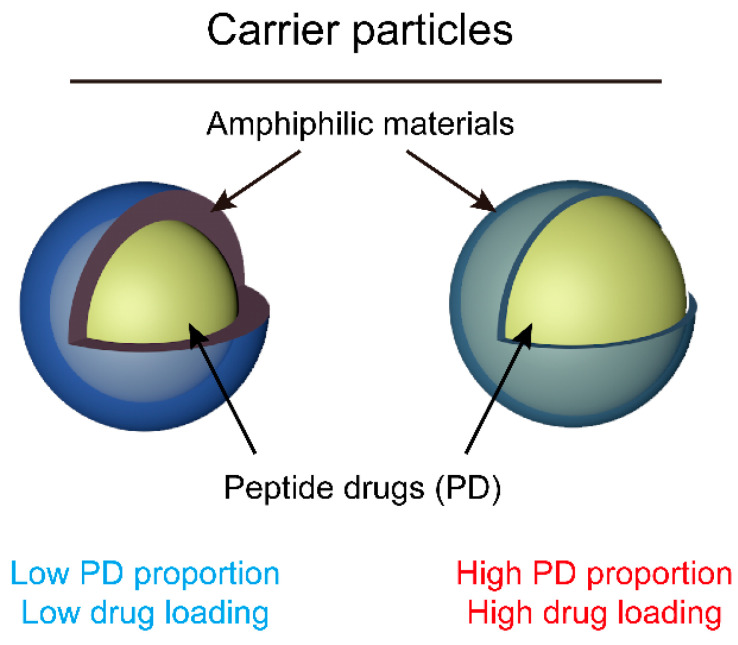
Comparison of low drug-loading and high drug-loading carrier particles.

According to the >90% content of amphiphilic materials in the carrier particles (Table 2), it could be reasonably inferred that amphiphilic materials occupied a very thick outer layer while peptide drugs accounted for a small inner zone. These particles can be viewed as ‘thick shell’ ones. A schematic illustration is depicted in Figure 1. Inspired by Figure 1, intriguingly, the ‘thick shell’ carrier particles (left panel) are associated with low drug loading, while the ‘thin shell’ carrier particles are associated with high drug loading. In order to enhance the drug loading, a ‘thinning of shell’ should be performed.

Herein, the authors propose several possible strategies to increase the proportion of peptide drugs to ‘thinning’ the ‘shell’ and fabricate ‘thin shell’ carrier particles, and ultimately enhance the drug loading, namely ‘physical thinning’, ‘chemical thinning’, and ‘leaving out’. The relevant considerations are also discussed.

### 4.1. Strategy 1: ‘Physical Thinning’

The first strategy, ‘physical thinning,’ means screening amphiphilic materials that have high surface-active properties and can form strong interaction with the peptide drugs.

(1) Methodology: Employing amphiphilic materials with high surface-active properties in the formulation of peptide drugs-containing MDIs;
(2) Principle: the high surface-active property enables the carrier particles to self-assemble at a low concentration [38] and generates merely a thin layer of amphiphilic materials; the strong interaction with the peptide drugs protects the latter from escaping the thin layer to cause conformational instability and therefore enables more peptide drugs to be encapsulated in the carrier particles;
(3) Examples: endogenous phospholipids in the lung region such as 1,2-dipalmitoyl-sn-glycero-3-phosphocholine (DPPC) had high surface-active properties and were proved to show intense interactions with peptide drugs [39,40].
(4) Advantages: With high surface-active properties, the content of amphiphilic materials can be controlled to a remarkably low level. Thus, not only the proportion of peptide drugs is elevated, but also the potential toxicity of amphiphilic materials will be minimized [41].
(5) Limitations: much attention should be paid to the selection of amphiphilic materials, as those with high surface-active properties may not have been officially approved for pharmaceutical uses.

### 4.2. Strategy 2: ‘Chemical Thinning’

The second strategy, ‘chemical thinning’, is defined as chemically linking the peptide drugs to the amphiphilic materials.

(1) Methodology: utilizing chemical reactions to bind peptide drugs with amphiphilic materials, and the products can self-assemble into carrier particles;
(2) Principle: There are many chemically active groups in peptide drugs that can be covalently bonded to the amphiphilic materials, and the conjugate may still possess the self-assemble attributes. Compared with physical encapsulation, chemical linking guarantees the stoichiometry between amphiphilic materials and peptide drugs [42], which will remarkably enhance the drug loading. Notably, if the molecule of a certain peptide drug is large enough, it can be linked to a hydrophobic chain instead of an amphiphilic material since the peptide drug per se can serve as the hydrophilic domain;
(3) Examples: functional polymers derived from poly(jasmine lactone) were chemically linked to the model drug doxorubicin [43]; chitosan-antimicrobial peptide conjugates were synthesized [44];
(4) Advantages: The physical stability of carrier particles can be substantially improved since the linked amphiphilic materials serve as a more solid ‘buffer zone’ compared with the conventional ones. They will not easily dissociate during storage to block the propellants;
(5) Limitations: chemical reactions may introduce unpredictable byproducts into the MDI system, adding burden to the quality-control process and even provoking potential toxicities.

### 4.3. Strategy 3: ‘Leaving out’

The third strategy, ‘leaving out’, is interpreted as omitting the amphiphilic materials and optimizing the cosolvent for dispersing peptide drugs.

(1) Methodology: screening a cosolvent where peptide drugs aggregate, and meanwhile, the physical stability is satisfactory;
(2) Principle: Theoretically, directly dispersing peptide drugs in the propellant without carrier particles will give a peptide drug proportion of 100%. Provided the peptide drugs can be stably dispersed in the propellant with the help of proper cosolvents, the drug loading may be significantly improved. If a certain peptide drug can form self-assembled objects, this strategy may be more applicable;
(3) Examples: cineole and n-heptane could construct a cosolvent with HFA 134a and exhibited great dispersing effects for model peptide drug lysozyme [45]; mitochondria-specific peptide amphiphiles with amphiphilic or lipidated modifications [12,13] could self-assemble into stable aggregates;
(4) Advantages: The theoretical drug loading can reach 100% without the addition of amphiphilic materials. In other words, the drug loading of peptide drugs-containing MDIs can be maximized by this strategy. It is especially orchestrated for those peptide drugs of large dosage;
(5) Limitations: There may be difficulties in properly finding a facile cosolvent for peptide drugs. In addition, the cosolvent generally contains organic solvents that can exert safety concerns.

## 5. Implications and Future Perspectives

The above three strategies are theoretically feasible, yet further experimental tests and R&D practices need to be performed to examine and optimize them. Specifically, for strategy 1, it is necessary to synthesize novel amphiphilic materials with high surface-active particles and accelerate their official approval. For strategy 2, green and economical synthesis route should be designed to link the peptide drugs to the amphiphilic materials. For strategy 3, efforts should be made to screen the appropriate cosolvent for peptide drugs aggregate. Multidisciplinary investigations need to be conducted to fulfill these preconditions before practically utilizing the strategies.

For different peptide drugs, the feasibility of these strategies may vary. For instance, peptide drugs with more hydrogen bonding sites have stronger interaction with amphiphilic materials, and hence the working concentration of amphiphilic materials employed in strategy 1 will be further reduced. Peptide drugs with more highly reactive functional groups will be easier to conjugate with amphiphilic materials, facilitating the application of strategy 2. Peptide drugs with higher tendency to form stable self-aggregate are more suitable for strategy 3. It is advisable to choose a tailored strategy for a specific peptide drug.

In addition, there are some general concerns during the application of the strategies. It should be borne in mind that the incompatibility between propellant and peptide drugs always confines the application scope of these strategies, and the instability provoked by the incompatibility should be strictly avoided during R&D for quality control purposes. Herein, the term ‘instability’ includes physical instability and chemical instability [46]. The sedimentation or layering of MDIs and the chemical degradation of peptide drugs should be prevented. From this standpoint, the molecular weight of amphiphilic materials and the release pattern of peptide drugs may exert impacts on the instability. On the one hand, amphiphilic materials with too low or too high molecular weight may have unsatisfactory miscibility with peptide drugs [47], and thus it will be difficult to construct an effective ‘buffer zone’. On the other hand, a premature release of peptide drugs into the bulk propellant will lead to the sedimentation and layering phenomena. In addition, one must consider the possibility of large-scale production when selecting a strategy for a targeted peptide drug [48].

Noticeably, some peptide drugs which require a lower dosage (such as exenatide in Table 3 and other toxin-derived peptide drugs) [49] can be found on the market. The low drug-loading problem may not be directly confronted if these peptide drugs are prepared into MDIs. Nonetheless, even for them, developing a formulation with higher drug-loading potential will be beneficial to assure the system’s stability to prevent the precipitation of drugs [50].

## 6. Summary and Concluding Remarks

Theoretically, MDIs exhibit a high potential for peptide drugs delivery. Nevertheless, due to the incompatibility between the propellant in MDIs formulation and peptide drugs, the drug-loading level of peptide drugs-containing MDI developed in previous studies could not well fulfill the treatment requirement in terms of the regular dosage of peptide drugs of interest. As a result, the applicability in clinical practices is impeded and, in turn, weakens the driving forces towards the industrialization process.

It is argued by the authors that enhancing the drug loading is the prerequisite to promoting the clinical translation of related products, and the current techniques cannot achieve this aim. Employing amphiphilic materials with high surface-active particles (‘physical thinning’), utilizing chemical reactions to bind peptide drugs with amphiphilic materials (‘chemical thinning’), and screening a cosolvent for peptide drug aggregates (‘leaving out’) are three possible potential methods to enhance the drug loading, put forward by the authors.

In future studies, the authors will test the applicability of the aforementioned three strategies. The authors also encourage the pharmaceutical industry to exploit innovative approaches, not limited to the three discussed here, to improve the drug loading of peptide drugs-containing MDIs. It is anticipated that relevant products can earlier achieve clinical translation and offer new remedies to diverse diseases.

## Figures and Tables

**Table 1:** Summary of basic information for HFA 134a and HFA 227. Adapted from Refs. [22,23].

Propellant	Molecular Formula	Molecular Weight	Boiling Point (°C)	Density (g/mL)	Solubility in Water (20 °C)
HFA 134a	C_2_H_2_F_4_	102.03	−26.5	1.210	1 in 1294 parts of water
HFA 227	C_3_HF_7_	170.03	−16.4	1.409	1 in 1725 parts of water

**Table 2:** Summary of previous studies on carrier particle encapsulation strategy.

Peptide Drugs	Amphiphilic Carrier	Propellant	The Drug Loading (mg/mL)	The Delivered Dose Per Spray (μg) ^a^	The Proportion of Amphiphilic Carrier (%) ^b^	Reference
Bovine serum albumin	PLGA	HFA 227	0.047	1.6~4.7	97.65	[27]
Exenatide	Poloxamer/ethanol	HFA 134a	0.103	3.6~10.3	93.50	[28]
Salmon calcitonin	Poloxamer/ethanol	HFA 134a	0.115	4.0~11.5	95.71	[29]
Insulin	Lecithin/lactose	HFA 134a	0.396	13.9~39.6	96.00	[33]
Insulin	Lecithin/lactose/glyceryl monooleate	HFA 134a	0.361	12.6~36.1	97.42	[31]
Salmon calcitonin	Lecithin	HFA 134a	0.171	6.0~17.1	91.14	[34]

^a^ Conversion based on the clinically available quantitative valve for pMDI (35, 50, and 100 μL). ^b^ Conversion according to the reference’s formulation details.

**Table 3:** Conventional dosage of peptide drugs commonly used in medicine.

Peptide Drugs	Indication	The Dose (Per Day or Per Use) (μg) ^a^	Reference
Thymopentin	Immunodeficiency disease	500	[51]
Insulin	Type I/type II diabetes	385	[52]
Mifamurtide	Osteosarcoma	285	[53]
Leuprorelin	Central precocious puberty	250	[54]
Ganirelix	Prevent premature ovulation	250	[55]
Octreotide	Stress ulcer and gastrointestinal bleeding	100	[56]
Salmon calcitonin	Osteoporosis	40	[57]
Oxytocin	Uterine bleeding caused by weak or poor contractions	25	[58]
Exenatide	Type II diabetes	10	[59]

^a^ According to the dosage recorded in the reference: If the peptide drug was administered once a few days, it was converted into the dose per day; if the peptide drug was administered multiple times a day, it was converted into the dose per use.

## Data Availability

Not applicable.
